# Dietary Energy–Protein Balance Regulates Slaughter Traits, Meat Quality, Digestive Enzyme Activity, and Fecal Microbiota in Early-Weaned Gray-Feathered King Pigeon Squabs

**DOI:** 10.3390/life16081273

**Published:** 2026-07-31

**Authors:** Yuanhao Li, Xiaobin Li, Huiguo Yang, Haiying Li, Yafei Liang, Mingcong Ding, Aikemu Maimaitijiang, Jie Ren, Honglei Sun, Jiajia Liu

**Affiliations:** 1 College of Animal Science, Xinjiang Agricultural University, Urumqi 830052, China; 17769614800@163.com (Y.L.); lxb262819@163.com (X.L.);; 2 Xinjiang Academy of Animal Husbandry Sciences, Urumqi 830011, China; 3 Moyu County Lanhai Pigeon Industry Development Co., Ltd., Hotan 848100, China

**Keywords:** gray-feathered King pigeon, metabolizable energy, crude protein, slaughter performance, meat quality, digestive enzyme activity, fecal microbiota

## Abstract

Early-weaned squabs are sensitive to dietary energy–protein supply, but combined effects remain unclear. We tested metabolizable energy (ME; 11.9, 12.2, and 12.5 MJ/kg) and crude protein (CP; 18, 19, and 20%) in 216 15-day-old gray-feathered King pigeon (*Columba livia domestica*) squabs using a 3 × 3 factorial design. Higher ME increased abdominal fat percentage, whereas CP affected slaughter traits. ME × CP interactions occurred for live weight, liver index, breast and leg muscle quality, and most digestive enzyme activities. At 18% CP, live weight was significantly higher at 12.5 than at 12.2 MJ/kg ME, whereas the opposite numerical trend occurred at 19% CP. Liver index was lower at 18% CP + 11.9 MJ/kg ME than at 18% CP + 12.2 MJ/kg ME. At 12.5 MJ/kg ME, breast muscle a* was lower at 20% CP than at 18% or 19% CP. Enzyme activities were generally highest at 20% CP + 11.9 MJ/kg ME. Targeted 16S rRNA sequencing associated the highest-energy/lowest-protein treatment with *Lactobacillus*-related taxa, whereas the opposite treatment showed higher diversity and greater contributions of Pseudomonadota- and Enterobacterales-related taxa. Responses were trait-specific; microbial findings represent associations between two contrasting diets.

## 1. Introduction

Meat pigeons are an important component of China’s specialty poultry industry, and early nutritional supply in squabs is closely associated with digestive organ development, nutrient utilization, and the formation of edible tissues after slaughter. During the early posthatch period, squabs obtain nutrients and microbial sources from pigeon crop milk. After early weaning, by contrast, their nutrient supply shifts from crop milk to formulated diets, requiring adaptive changes in both the digestive system and intestinal microbiota [1]. Therefore, the adequacy of dietary nutrient structure during early weaning may influence not only nutrient digestion but also slaughter performance, meat quality, and intestinal microbial stability.

Dietary metabolizable energy (ME) and crude protein (CP) are among the most critical nutritional factors in poultry diet formulation, jointly determining energy supply, amino acid utilization, tissue deposition, and fat accumulation. Insufficient ME supply may restrict nutrient deposition and tissue development, whereas excessive energy intake may increase the risk of fat deposition. Dietary CP level affects protein deposition and amino acid availability and may also alter the pool of nitrogenous substrates available in the intestine. Previous poultry studies have shown that ME and CP levels can jointly affect muscle composition, carcass traits, meat quality, and intestinal microbial structure [2,3,4], indicating that energy–protein balance may explain the integrated physiological response to diet more effectively than either nutrient factor alone.

Slaughter performance, organ index, meat quality, and digestive enzyme activity reflect the responses of early-weaned squabs to dietary energy–protein combinations from different physiological perspectives. Slaughter performance reflects the conversion of nutrient supply into edible tissue deposition, whereas meat quality is closely related to muscle metabolism, postmortem acidification, meat color, and water loss rate [5,6]. In parallel, small-intestinal digestive enzyme activity directly indicates the capacity to hydrolyze proteins, lipids, and starch. The intestinal microbiota participates in nutrient degradation, metabolite production, barrier maintenance, and host immune regulation, and thus represents a key link between dietary nutrient structure and host physiological responses [7,8]. Jointly assessing slaughter performance, meat quality, digestive enzyme activity, and fecal microbiota can therefore provide a more comprehensive evaluation of the adaptive characteristics of early-weaned squabs under different energy–protein combinations.

Current studies on the nutrient requirements of meat pigeons have focused mainly on parental feeding systems or conventional production stages, whereas the combined effects of ME and CP in early-weaned gray-feathered King pigeon squabs remain insufficiently evaluated. Previous pigeon studies have examined dietary ME values of approximately 12.2–12.8 MJ/kg and CP levels up to approximately 18% under breeder-feeding systems and conventional production conditions [9,10]. Because specific recommendations for early-weaned squabs consuming complete pelleted diets remain limited, the present ME range of 11.9–12.5 MJ/kg was selected to bracket a practically relevant energy supply, while the 18–20% CP range extended the protein gradient for this rapid-growth and digestive-adaptation stage. In particular, few studies have assessed slaughter performance, organ index, breast and leg muscle quality, intestinal digestive enzyme activity, and fecal microbial community structure within the same experimental system. Accordingly, the present study used a 3 × 3 factorial design to evaluate the main and interactive effects of dietary ME and CP on these outcomes. We hypothesized that ME and CP would exert trait-specific main and interactive effects on carcass traits, organ indices, meat quality, and digestive enzyme activity, and that two a priori selected opposite energy–protein combinations would be associated with distinct fecal microbial community structures.

## 2. Materials and Methods

### 2.1. Ethics Approval Statement

The animal study protocol was approved by the Ethics Committee for Laboratory Animals of the Institute of Animal Husbandry, Xinjiang Academy of Animal Husbandry Sciences (Approval No. [2026] 02; approval date: 23 March 2026).

### 2.2. Experimental Design

A total of 216 healthy 15-day-old gray-feathered King pigeon squabs with similar body weights were selected for a 3 × 3 factorial experiment. The two experimental factors were three ME levels (11.9, 12.2, and 12.5 MJ/kg) and three CP levels (18%, 19%, and 20%). These levels were determined based on previous reports and preliminary trials conducted by the research team, and were selected to cover a practically relevant range for nutritional regulation in early-weaned squabs. The squabs were randomly assigned to nine dietary treatments, with eight replicates per treatment and three birds per replicate. The feeding trial lasted 28 days. All squabs were provided by Moyu County Lanhai Pigeon Industry Development Co., Ltd., and the breed was American gray-feathered King pigeon (*Columba livia domestica*).

### 2.3. Feeding and Routine Management

The experiment was conducted at Moyu County Lanhai Pigeon Industry Development Co., Ltd., Hotan, Xinjiang, China. Before the trial, the pigeon house was thoroughly washed and disinfected, and the squabs were vaccinated according to the farm immunization program. During the trial, the house temperature ranged from 20 to 34 °C, with mean values of 29 to 31 °C, and relative humidity ranged from 46% to 62%, with mean values of 56% to 60%. The birds were housed in the middle tier of vertical cages, with three squabs per cage (45 × 50 × 60 cm). Birds were fed a complete pelleted diet that was offered manually at 10:00 each day and withdrawn at 21:00 each evening; therefore, feed access was scheduled rather than continuous. Feeders were checked four times daily to ensure feed availability during the feeding period. Water was available ad libitum throughout the trial, and drinkers were inspected daily to remove feces or debris and maintain water quality. Supplemental lighting was adjusted according to bird age, and natural ventilation was combined with positive-pressure ventilation. Feeding management was consistent with routine farm practice. Diets were formulated according to the Tables of Feed Composition and Nutritive Values in China (35th edition) [11], and the ingredient composition and calculated nutrient levels are shown in Table 1.

### 2.4. Slaughter Performance

At the end of the trial, feed was withdrawn at 21:00 on the evening before slaughter in accordance with the routine nightly feeding schedule, while water remained available ad libitum. One healthy bird with a body weight close to the replicate mean was selected from each replicate for slaughter performance measurement, resulting in eight birds per treatment. Live body weight was recorded individually before slaughter. After slaughter, exsanguination, and defeathering, carcass dissection and weighing were performed according to NY/T 823-2020, Performance Terminology and Measurement Calculation Methods of Poultry Production [12]. The measured traits included carcass weight, half-eviscerated carcass weight, eviscerated carcass weight, breast muscle weight, leg muscle weight, and abdominal fat weight. Abdominal fat included visible fat in the abdominal cavity and fat surrounding the gizzard. Slaughter yield, half-carcass yield, full-carcass yield, breast muscle ratio, leg muscle ratio, and abdominal fat percentage were calculated according to the corresponding formulas in the standard and expressed as percentages.

### 2.5. Organ Index

After slaughter and dissection, the liver, spleen, proventriculus, gizzard, and bursa of Fabricius were immediately collected. Residual blood, fat, and connective tissue were removed from the organ surfaces. The proventriculus and gizzard were opened, their contents were removed, and the tissues were gently rinsed with precooled 0.9% saline and blotted dry with filter paper. Fresh organ weights were measured using an electronic balance. Organ index was calculated as the fresh organ weight relative to preslaughter live body weight using the formula: organ index (%) = fresh organ weight/preslaughter live body weight × 100.

### 2.6. Meat Quality

Breast muscle was treated as the primary meat-quality tissue because of its major economic importance in meat pigeons, while leg muscle was evaluated as a supplementary tissue to identify muscle-specific responses. Visible fat, fascia, and connective tissue were removed before analysis. The cut surface was exposed to air for a consistent period, after which lightness (L*), redness (a*), and yellowness (b*) were measured at three positions using an NR110 portable precision colorimeter (Shenzhen Threenh Technology Co., Ltd., Shenzhen, China; 4 mm aperture; d/8° geometry); the mean value was used for analysis. Muscle pH was measured using a calibrated pH-Star portable pH meter (Matthäus, Germany), with the electrode inserted into the central portion of the muscle while avoiding fascia and fat. Water loss rate was measured by the pressing method: samples of uniform size were weighed (W1), pressed under a 35 kg load for 5 min, and reweighed (W2); water loss rate (%) was calculated as (W1 − W2)/W1 × 100. Shear force was measured perpendicular to the muscle-fiber direction using a C-LM3B tenderness meter (Tenovo, Beijing, China). Each measurement was performed in triplicate, and the mean was used for statistical analysis.

### 2.7. Intestinal Digestive Enzyme Activity

After slaughter, the duodenum and jejunum were rapidly excised, and tissue samples were collected from standardized anatomical locations. Intestinal contents were removed, and the tissues were gently rinsed with precooled 0.9% saline, blotted dry, snap-frozen in liquid nitrogen, and stored at −80 °C. Before measurement, intestinal tissue was weighed and homogenized in precooled homogenization medium according to the kit instructions. The homogenates were centrifuged, and the supernatants were used to determine trypsin, chymotrypsin, lipase, and α-amylase activities. All four outcomes were measured as enzyme activities using commercial microplate-based biochemical activity assay kits obtained from Jiangsu Meimian Industrial Co., Ltd. (Yancheng, China) and Jiangsu Aidisheng Biological Technology Co., Ltd. (Yancheng, China). The archived records did not preserve catalog numbers or a definitive analyte-by-supplier assignment; therefore, those details are not reported. The α-amylase assay used a starch–iodine colorimetric principle, and the remaining enzyme activities were quantified using the corresponding microplate protocols specified by the manufacturers. No cytokines, immunoglobulins, hormones, or other ELISA-based analytes were measured in this section. Enzyme activities were calculated in the units specified by the respective kits and normalized to tissue protein where required.

### 2.8. Fecal 16S rRNA Gene Amplicon Sequencing and Bioinformatics Analysis

Two contrasting dietary treatments were selected a priori for targeted fecal 16S rRNA gene amplicon sequencing: R1 (18% CP + 12.5 MJ/kg ME) and R2 (20% CP + 11.9 MJ/kg ME), with eight biological replicates per treatment. R1 represented the highest-energy/lowest-protein combination, whereas R2 represented the lowest-energy/highest-protein combination in the 3 × 3 factorial design. Because the two treatments occupied opposite corners of the design and provided the greatest contrast in dietary energy–protein supply, they were selected to compare fecal microbial community structure under two contrasting nutritional patterns rather than to provide a complete factorial microbiota assessment. The selection was made before the phenotypic results were available. Fresh fecal samples were collected from designated birds in the selected treatments using sterile sampling tubes. These birds were not the same individuals subsequently selected for slaughter measurements. Samples were immediately placed on ice, transported to the laboratory, snap-frozen in liquid nitrogen, and stored at −80 °C until DNA extraction.

Total microbial DNA was extracted from fecal samples by Novogene Co., Ltd. (Beijing, China) using its standardized fecal microbial DNA extraction workflow. The extraction-kit model was not retained in the archived service documentation and therefore could not be verified. The concentration and purity of the extracted DNA were assessed spectrophotometrically, and DNA integrity was evaluated by agarose gel electrophoresis. Only DNA samples that met the sequencing provider’s quality-control criteria were used for PCR amplification. The bacterial 16S rRNA V4 region was amplified using primers 515F and 806R. Each PCR mixture contained 15 μL of Phusion High-Fidelity PCR Master Mix, 0.2 μM of each primer, and 10 ng of genomic DNA. Thermal cycling consisted of initial denaturation at 98 °C for 1 min; 30 cycles of 98 °C for 10 s, 50 °C for 30 s, and 72 °C for 30 s; and a final extension at 72 °C for 5 min. PCR products were purified using magnetic beads and pooled in equimolar amounts. Libraries were quantified using Qubit and qPCR and sequenced on an Illumina NovaSeq 6000 platform.

Raw reads were demultiplexed according to barcode sequences, and barcode and primer sequences were removed. Paired-end reads were merged using FLASH version 1.2.11, residual reverse-primer sequences were removed using Cutadapt version 3.3, and read quality was filtered using fastp version 0.23.1. Chimeric sequences were identified against the SILVA reference database and removed using VSEARCH version 2.16.0. Denoising and amplicon sequence variant (ASV) inference were performed using the DADA2 plugin in QIIME 2 release 2025.4. Taxonomic annotation was conducted against SILVA 138.1, and samples were rarefied to the minimum retained sequencing depth before diversity analyses. Alpha diversity was assessed using Shannon, Simpson, Pielou_e, Chao1, and observed-features indices. Beta diversity was evaluated using Bray–Curtis distance, followed by principal coordinate analysis (PCoA) and analysis of similarities (ANOSIM) in R version 4.0.3. Differential taxa were identified using linear discriminant analysis effect size (LEfSe) version 1.1.01, with an LDA score threshold of 4.0.

### 2.9. Statistical Analysis

Data were organized using WPS Excel 2025 and analyzed in SPSS 27.0. Before inferential analysis, the normality of the residuals was assessed using the Shapiro–Wilk test, and homogeneity of variances was evaluated using Levene’s test. A two-way analysis of variance was performed using the general linear model procedure to test the fixed effects of dietary ME level, CP level, and their interaction. Each replicate cage was used as the experimental unit, and results are expressed as mean ± standard error of the mean (SEM). When significant differences were detected, Duncan’s multiple range test was used for post hoc comparisons. To maintain the factorial interpretation, superscripts among the nine treatment combinations were presented only for variables with a significant ME × CP interaction. When the interaction was nonsignificant, combination-level superscripts were omitted, and superscripts on marginal means were retained only when the corresponding main effect was significant. Differences were considered significant at *p* < 0.05 and highly significant at *p* < 0.01.

For the 16S rRNA gene amplicon sequencing data, alpha diversity indices between the R1 and R2 groups were compared using an independent-samples *t*-test. Beta-diversity differences were evaluated by analysis of similarities (ANOSIM) based on Bray–Curtis distances, and differential taxa were identified by linear discriminant analysis effect size (LEfSe) version 1.1.01 using a linear discriminant analysis (LDA) score threshold of 4.0.

## 3. Results

### 3.1. Slaughter Performance

As shown in Table 2, dietary ME significantly affected abdominal fat percentage (*p* = 0.020), whereas dietary CP affected slaughter yield, half-carcass yield, full-carcass yield, leg muscle ratio, and abdominal fat percentage (*p* < 0.05). A significant ME × CP interaction was observed for live weight (*p* = 0.013). At 18% CP, live weight was significantly higher at 12.5 than at 12.2 MJ/kg ME (*p* < 0.05). At 19% CP, the numerical trend was reversed, with lower live weight at 12.5 than at 12.2 MJ/kg ME; at 12.5 MJ/kg ME, live weight was lower at 19% than at 18% CP (*p* < 0.05). Thus, the live-weight response to ME depended on CP level. No significant interaction was observed for the remaining slaughter traits (*p* > 0.05).

### 3.2. Organ Index

As shown in Table 3, dietary ME and CP had a significant interaction effect on liver index (*p* = 0.006). At 18% CP, the liver index was lower at 11.9 than at 12.2 MJ/kg ME (*p* < 0.05), with the 12.5 MJ/kg ME value intermediate. At 11.9 MJ/kg ME, the liver index was lower at 18% CP than at 19% or 20% CP (*p* < 0.05). Neither the main effects of ME or CP nor their interaction significantly affected spleen index, proventriculus index, gizzard index, or bursa of Fabricius index (*p* > 0.05); therefore, no significance letters or pairwise claims are presented for these outcomes.

### 3.3. Breast Muscle Quality

As shown in Table 4, dietary ME and CP had significant interaction effects on breast muscle a*, b*, and pH (*p* < 0.05). The original treatment-combination superscripts were therefore retained for these outcomes. For a*, the 20% CP + 12.5 MJ/kg ME treatment was lower than the 18% and 19% CP treatments at the same ME level and lower than the other ME levels within 20% CP (*p* < 0.05). For b*, the 19% CP treatment was lower than the 18% and 20% CP treatments at 11.9 MJ/kg ME, while within 20% CP the 12.5 MJ/kg ME value was lower than the 11.9 MJ/kg ME value (*p* < 0.05). For pH, the 18% CP treatment was lower than the 19% and 20% CP treatments at 11.9 MJ/kg ME; at 12.2 MJ/kg ME, the 20% CP value was lower than the 19% CP value, with 18% CP intermediate (*p* < 0.05). Breast muscle water loss rate, shear force, and L* were not significantly affected by ME, CP, or their interaction (*p* > 0.05).

### 3.4. Leg Muscle Quality

As shown in Table 5, dietary ME and CP had a significant interaction effect on leg muscle pH (*p* = 0.015). At 11.9 MJ/kg ME, pH was higher at 20% CP than at 18% or 19% CP (*p* < 0.05). Within 20% CP, pH was higher at 11.9 than at 12.2 or 12.5 MJ/kg ME (*p* < 0.05). Leg muscle water loss rate, shear force, L*, a*, and b* were not significantly affected by either main effect or by the ME × CP interaction (*p* > 0.05); therefore, no significance letters or isolated pairwise claims are presented for these outcomes.

### 3.5. Intestinal Digestive Enzyme Activity

As shown in Table 6, significant ME × CP interactions were observed for duodenal trypsin, lipase, and α-amylase activities and for jejunal trypsin, chymotrypsin, lipase, and α-amylase activities (*p* < 0.05). The original treatment-combination superscripts were retained for these interaction-responsive outcomes. Although response patterns varied among enzymes, activities were generally high in the 20% CP + 11.9 MJ/kg ME treatment and low in the 18% CP + 12.5 MJ/kg ME treatment. The interaction was nonsignificant for duodenal chymotrypsin activity (*p* = 0.725); its marginal means decreased with increasing ME and increased with increasing CP (*p* < 0.001). These measurements describe tissue enzyme activities and do not establish increased secretion.

### 3.6. Fecal 16S rRNA Gene Amplicon Data Analysis

The R1 (18% CP + 12.5 MJ/kg ME) and R2 (20% CP + 11.9 MJ/kg ME) treatments were selected a priori as opposite-corner dietary combinations representing the highest-energy/lowest-protein and lowest-energy/highest-protein conditions, respectively. This targeted comparison was intended to maximize contrast in dietary energy–protein supply; it was not selected retrospectively from slaughter, meat-quality, or digestive-enzyme results and does not represent a full 3 × 3 factorial microbiota analysis.

#### 3.6.1. Alpha Diversity and Amplicon Sequence Variant Distribution

To evaluate the effects of different energy–protein combinations on fecal microbial alpha diversity in gray-feathered King pigeon squabs, the Shannon, Simpson, Pielou_e, Chao1, and observed features indices were compared between the R1 and R2 groups (Table 7). The Shannon, Simpson, and Pielou_e indices were significantly higher in the R2 group than in the R1 group (*p* < 0.01), indicating greater fecal microbial diversity and evenness in the R2 group. In contrast, the Chao1 index and observed features were numerically higher in the R2 group than in the R1 group, but the differences were not statistically significant (*p* > 0.05). These results suggested that the number of detectable microbial taxa tended to increase under this treatment, whereas the difference in community richness was relatively limited.

**Figure 1:**
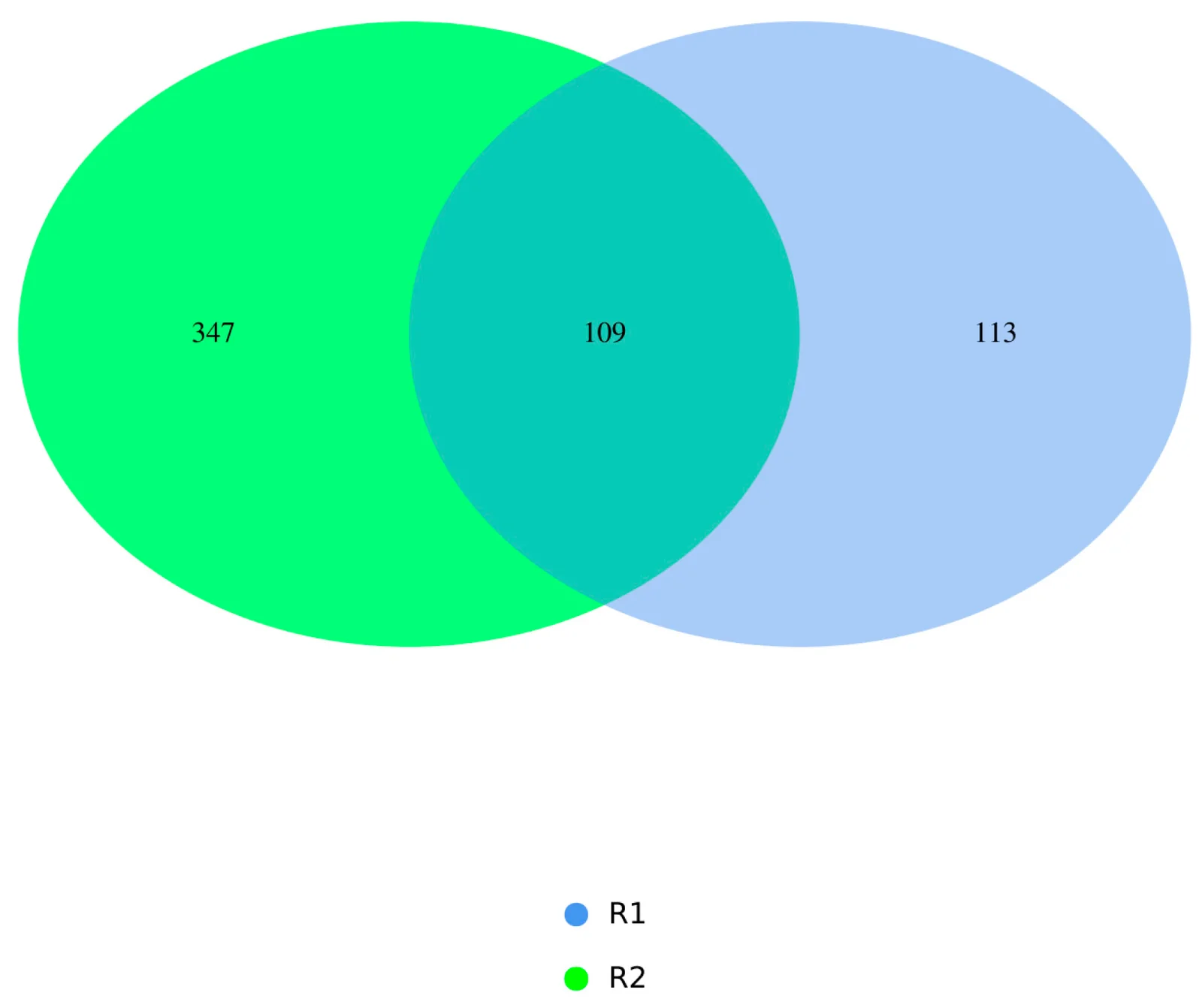
Shared and unique ASVs of fecal microbiota in the R1 and R2 groups. R1, 18% CP + 12.5 MJ/kg ME; R2, 20% CP + 11.9 MJ/kg ME; ME, metabolizable energy; CP, crude protein; ASV, amplicon sequence variant.

Venn analysis further characterized the shared and unique ASV composition of the R1 and R2 groups (Figure 1). A total of 109 ASVs were shared between the two groups, indicating that a common core microbiota persisted in gray-feathered King pigeon squabs under the two nutritional combinations. The R1 group contained 113 unique ASVs, whereas the R2 group contained 347 unique ASVs, suggesting that the R2 group had more treatment-specific ASVs and potentially greater compositional heterogeneity. It should be noted that a Venn diagram reflects the presence or absence of ASVs and their shared relationships, rather than differences in relative abundance; therefore, these results should be interpreted together with community composition and differential taxa analyses.

#### 3.6.2. Beta Diversity Based on Principal Coordinate Analysis and Analysis of Similarities

**Figure 2:**
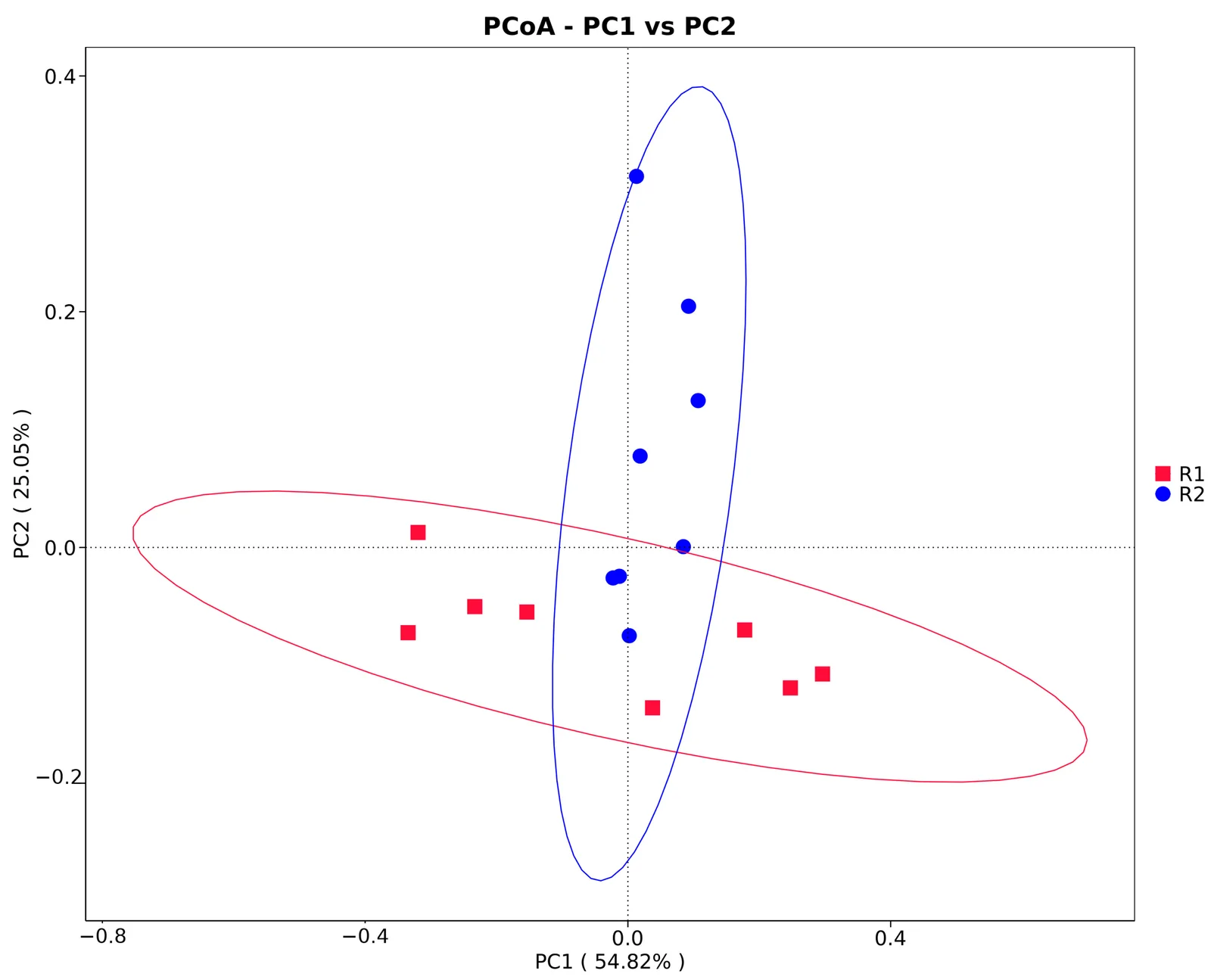
Principal coordinate analysis (PCoA) based on Bray–Curtis distance. The first and second principal coordinates explained 54.82% and 25.05% of the community variation, respectively. Red squares and blue circles represent individual R1 and R2 samples, respectively; the corresponding colored ellipses outline the group distributions, and the dotted lines indicate zero-coordinate reference lines. R1, 18% CP + 12.5 MJ/kg ME; R2, 20% CP + 11.9 MJ/kg ME; ME, metabolizable energy; CP, crude protein.

To further compare overall fecal microbial community structure between the R1 and R2 groups, principal coordinate analysis (PCoA) and analysis of similarities (ANOSIM) were performed based on Bray–Curtis distance (Figure 2). The PCoA results showed that the first principal coordinate (PC1) and second principal coordinate (PC2) explained 54.82% and 25.05% of the community variation, respectively, with the first two principal coordinates explaining 79.87% of the total variation. This indicated that the ordination captured the major differences in microbial community structure among samples. The sample distribution showed a degree of separation between the R1 and R2 groups in the principal coordinate space. The ANOSIM analysis further indicated a significant difference in community structure between the two groups (R = 0.24944, *p* = 0.015), suggesting that different energy–protein combinations affected the overall fecal microbial community structure of gray-feathered King pigeon squabs.

#### 3.6.3. Fecal Microbial Community Composition

**Figure 3:**
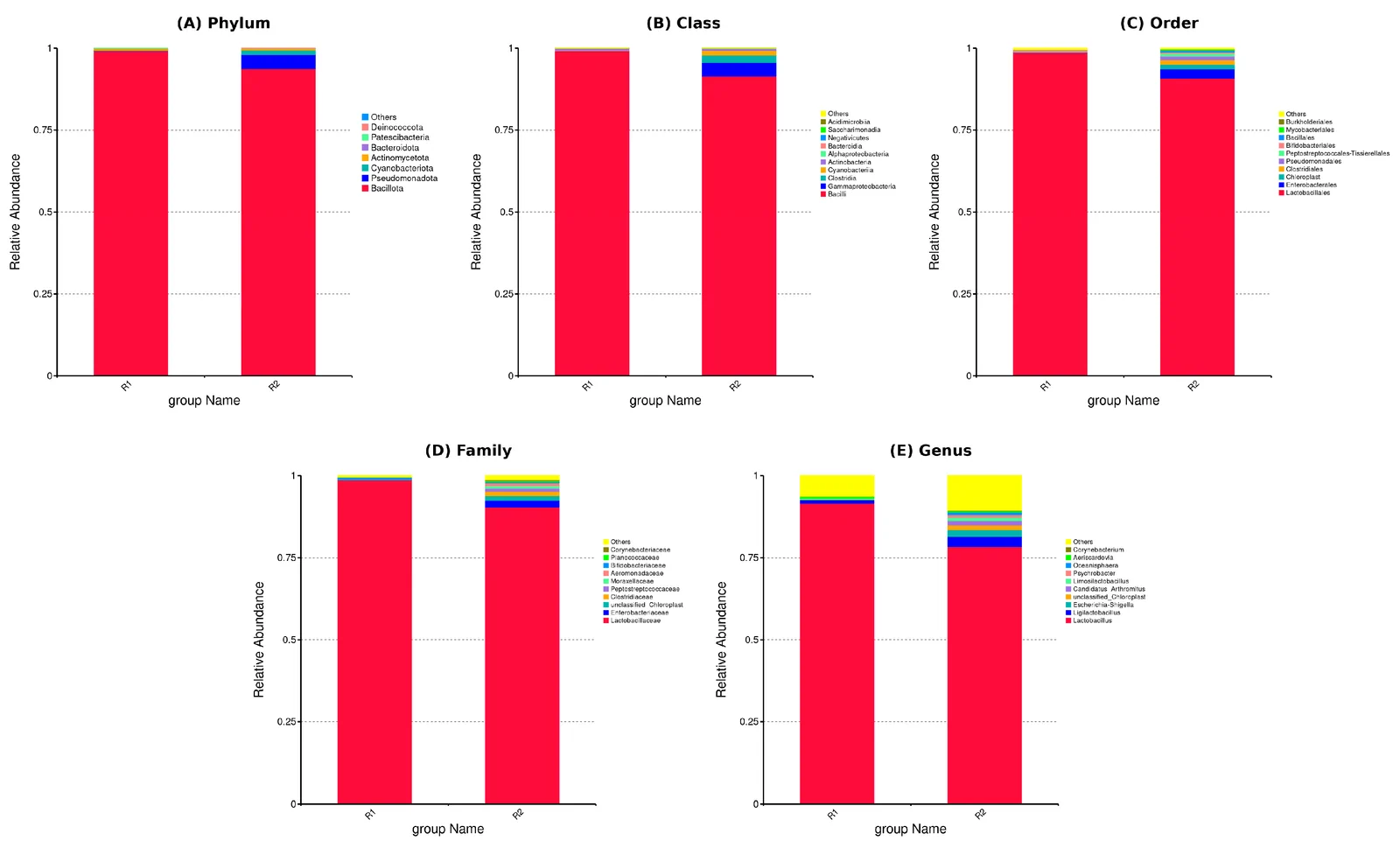
Relative abundance composition of fecal microbiota in the R1 and R2 groups at the phylum, class, order, family, and genus levels. R1, 18% CP + 12.5 MJ/kg ME; R2, 20% CP + 11.9 MJ/kg ME; ME, metabolizable energy; CP, crude protein.

Relative abundance analysis further revealed fecal microbial community composition in the R1 and R2 groups at different taxonomic levels (Figure 3). Overall, samples from both groups showed a community structure dominated by a limited number of abundant taxa, with multiple low-abundance taxa contributing to the remaining composition. Nevertheless, the relative proportions of dominant and low-abundance taxa differed between the two nutritional combinations. At the phylum level, Bacillota was the dominant phylum shared by both groups, indicating that Bacillota-related taxa were the main components of the fecal microbiota in gray-feathered King pigeon squabs. Compared with the R1 group, the R2 group showed a lower relative abundance of Bacillota and higher relative abundances of Pseudomonadota, Cyanobacteriota, and Actinomycetota.

At the class, order, and family levels, the differences between the two groups were mainly reflected in changes in the relative proportions of lactic acid bacteria-related taxa and Pseudomonadota-related taxa. The dominant taxa in the R1 group were concentrated in Bacilli, Lactobacillales, and Lactobacillaceae, indicating that the fecal microbial community in this group was more strongly centered on lactic acid bacteria-related taxa. In contrast, the R2 group showed higher relative abundances of Gammaproteobacteria, Enterobacterales, and Enterobacteriaceae, accompanied by increases in several other low-abundance taxa.

At the genus level, *Lactobacillus* was the dominant genus shared by both groups, but its relative abundance was higher in the R1 group than in the R2 group, suggesting that the R1 treatment may be more favorable for maintaining a *Lactobacillus-centered* fecal microbial structure. In contrast, the R2 group showed higher proportions of *Escherichia-Shigella*, *Ligilactobacillus*, *Candidatus_Arthromitus*, *Limosilactobacillus*, and several low-abundance genera, indicating a more dispersed fecal microbial composition and a greater contribution of low-abundance taxa to the overall community structure. Increased relative abundances of *Escherichia-Shigella* and Enterobacteriaceae-related taxa should not be directly equated with a pathological state. Rather, they suggest that the lower energy-higher protein combination may alter fermentable substrates and the microecological environment in the hindgut, thereby affecting the ecological niches of facultative anaerobes and related taxa.

#### 3.6.4. Differential Taxa Analysis Based on Linear Discriminant Analysis Effect Size

**Figure 4:**
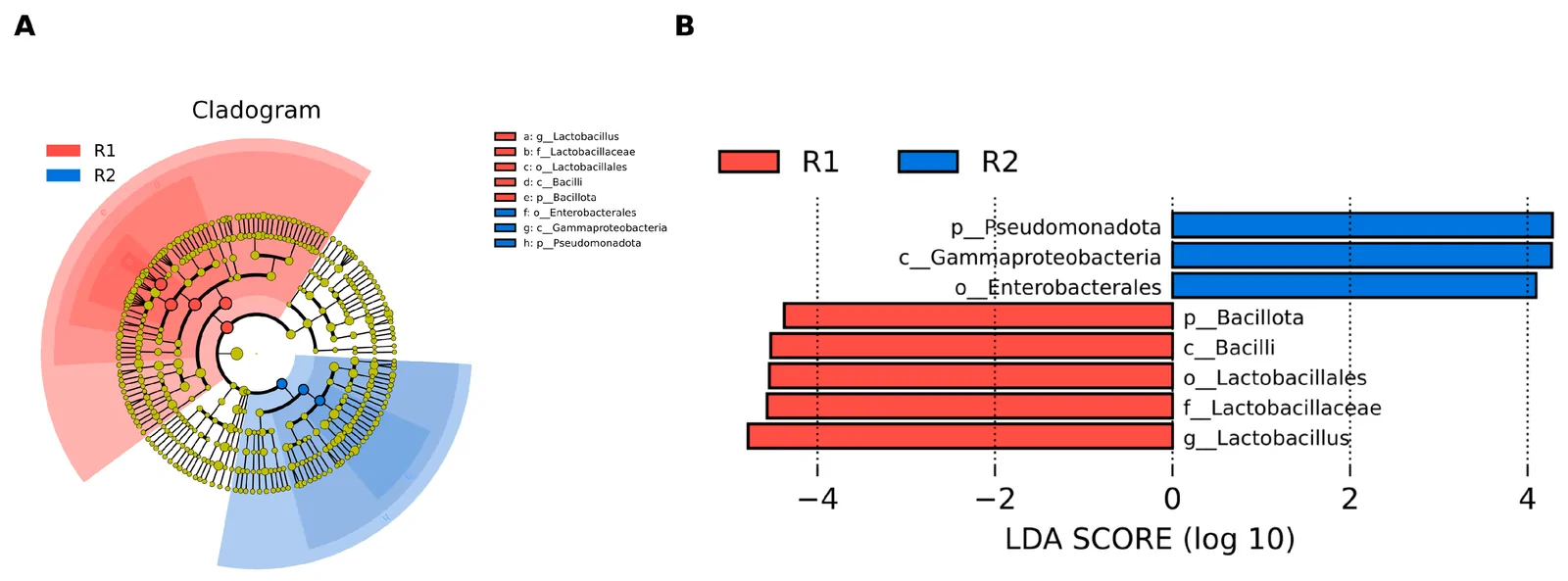
Differential fecal microbial taxa identified using linear discriminant analysis effect size (LEfSe) between the R1 and R2 groups. (**A**) Cladogram; (**B**) LDA score plot. R1, 18% CP + 12.5 MJ/kg ME; R2, 20% CP + 11.9 MJ/kg ME; ME, metabolizable energy; CP, crude protein. Only taxa with an LDA score ≥ 4.0 are shown.

Linear discriminant analysis effect size (LEfSe) analysis was used to identify discriminatory taxa between the R1 and R2 groups (Figure 4). The taxa enriched in the R1 group mainly included Bacillota, Bacilli, Lactobacillales, Lactobacillaceae, and *Lactobacillus*, forming a relatively continuous lactic acid bacteria-related taxonomic chain from phylum to genus. This result was consistent with the higher relative abundance of *Lactobacillus* observed in the R1 group in the relative abundance analysis, indicating that the between-group differences in the fecal microbial community of the R1 group were mainly attributable to lactic acid bacteria-related taxa.

By contrast, the characteristic taxa in the R2 group were mainly concentrated in Pseudomonadota, Gammaproteobacteria, and Enterobacterales. These taxa were consistent with the increased relative abundance of Pseudomonadota- and Enterobacterales-related taxa observed in the R2 group, suggesting a greater contribution of Pseudomonadota-related taxa to the between-group differences under the R2 treatment. Overall, the alpha diversity, ASV sharing patterns, PCoA/ANOSIM, relative abundance composition, and LEfSe results collectively indicated that the two energy–protein combinations produced distinct fecal microbial community structures in gray-feathered King pigeon squabs. The R1 group was characterized by a dominant community structure centered on *Lactobacillus* and its higher-level taxonomic lineages, whereas the R2 group showed increased diversity and evenness, a greater number of unique ASVs, and greater contributions of Pseudomonadota-, Enterobacterales-, and Enterobacteriaceae-related taxa.

## 4. Discussion

Slaughter performance is a core indicator of the conversion of dietary nutrients into edible tissue in meat pigeons [13]. In the present study, higher ME increased abdominal fat percentage, supporting the established role of energy supply in poultry fat deposition [14]. Dietary CP affected several carcass-yield traits, and 19% CP was associated with higher half-carcass and full-carcass yields than the other CP levels. Importantly, the significant ME × CP interaction for live weight (*p* = 0.013) showed that energy did not produce a uniform response across protein levels: increasing ME from 12.2 to 12.5 MJ/kg increased live weight at 18% CP but reduced it at 19% CP. This crossover pattern is biologically more informative than either main effect alone and indicates that live-weight responses should be interpreted in the context of the accompanying protein supply. However, because the interaction was not significant for the principal carcass-yield traits, the live-weight interaction should not be generalized as evidence that one combination optimized overall slaughter performance. Previous pigeon studies similarly reported stage- and feeding-pattern-dependent responses to dietary energy–protein balance [9,15,16,17].

Organ indices provide morphological evidence of relative organ development but do not directly measure organ function [18]. The liver index showed a significant ME × CP interaction, whereas the spleen, proventriculus, gizzard, and bursa of Fabricius indices remained statistically stable. The lowest liver index occurred in the 18% CP + 11.9 MJ/kg ME treatment, while higher values occurred under several other combinations, demonstrating a nonparallel response to energy across CP levels. Because neither liver biochemical function nor histopathology was assessed, this interaction should be interpreted as a difference in relative liver mass rather than proof of beneficial or adverse metabolic adaptation. The removal of unsupported spleen pairwise comparisons further aligns the biological interpretation with the factorial ANOVA results. Stage-dependent liver and digestive-organ development reported in pigeons [10,19,20] supports cautious interpretation of these relative-weight changes.

Breast muscle was considered the primary meat-quality tissue because it constitutes the principal edible and economically valuable muscle in meat-type pigeons. Leg muscle was retained as a supplementary tissue to determine whether responses were muscle-specific. In breast muscle, significant ME × CP interactions were confined to a*, b*, and pH, whereas water loss rate, shear force, and L* were stable. Treatment-combination comparisons showed that the most evident a* reduction occurred in the 20% CP + 12.5 MJ/kg ME treatment; b* was lower at 19% CP than at 18% or 20% CP under 11.9 MJ/kg ME; and pH responses differed across both nutrient backgrounds. These outcomes indicate that energy–protein combinations influenced color and postmortem acidification more clearly than tenderness or water loss. Because color and pH are influenced by muscle metabolism, pigments, and postmortem glycolysis [21,22,23,24,25], the observed differences are relevant to product appearance, but they do not establish a comprehensive improvement in breast-meat quality. The nonsignificant shear-force result also precludes attributing superior tenderness to any individual combination.

Leg muscle was analyzed as a supplementary parameter because its fiber composition, myoglobin content, and metabolic characteristics differ from those of breast muscle [23,26]. Only leg muscle pH showed a significant ME × CP interaction; water loss rate, shear force, L*, a*, and b* showed no significant main or interaction effects. At 11.9 MJ/kg ME, pH was higher at 20% CP than at 18% or 19% CP, whereas within 20% CP it was higher at 11.9 than at 12.2 or 12.5 MJ/kg ME. This combination-dependent response, together with the stability of the other traits, indicates that leg-muscle quality was less responsive than breast-muscle quality within the tested range. Unsupported pairwise findings from the former nine-group comparison were removed to avoid overstating the practical importance of leg-muscle differences.

Digestive enzyme activities describe the capacity of intestinal tissue homogenates to hydrolyze proteins, lipids, and starch under the assay conditions [27,28,29]. Specifically, trypsin and chymotrypsin reflect proteolytic capacity, lipase reflects lipid hydrolysis, and α-amylase reflects starch hydrolysis. The transition from crop milk, which is rich in proteins, lipids, and bioactive components, to formulated feed may further contribute to the developmental sensitivity of digestive function in early-weaned squabs [30,31]. Significant interactions for most enzymes show that the response to ME varied with CP level rather than being determined by a single nutrient factor. Activities were generally higher in the 20% CP + 11.9 MJ/kg ME treatment and lower in the 18% CP + 12.5 MJ/kg ME treatment. This pattern is compatible with an adaptive response to differences in dietary substrate load, but the present measurements cannot distinguish enzyme synthesis, luminal secretion, tissue retention, feed intake effects, or digestive efficiency. Therefore, higher activity should not be equated automatically with improved secretion or production performance. The practical interpretation must be integrated with live weight, carcass traits, and meat quality, none of which consistently identified the 20% CP + 11.9 MJ/kg ME combination as globally optimal [32,33,34].

Dietary composition can reshape poultry gut microbial ecology and thereby influence nutrient utilization, intestinal stability, and host responses [35,36,37]. The targeted microbiota comparison identified distinct fecal community patterns between the two a priori selected opposite-corner treatments. R2 showed higher Shannon, Simpson, and Pielou_e indices and more unique ASVs than R1, whereas richness estimates did not differ significantly. These findings indicate higher diversity and evenness under the lowest-energy/highest-protein condition but do not, by themselves, indicate better intestinal health. PCoA and ANOSIM supported community differentiation, although the modest ANOSIM effect size indicates partial restructuring rather than complete separation or dysbiosis [38,39,40]. Because the microbiota samples were obtained from different individuals from those used for slaughter and meat-quality measurements, direct individual-level correlations between microbial and phenotypic outcomes cannot be inferred.

Linear discriminant analysis effect size (LEfSe) identifies taxa with differential discriminatory contributions between groups [41]. Taxonomic composition and LEfSe analyses showed that R1 was characterized by *Lactobacillus*-related lineages, whereas R2 had greater contributions from Pseudomonadota-, Enterobacterales-, and Enterobacteriaceae-related taxa. These results are associations with contrasting dietary structures rather than evidence that specific taxa caused the host phenotypes. *Lactobacillus*-related enrichment may be consistent with a community more strongly dominated by lactic-acid-bacteria lineages [42,43], while the increased contribution of facultative anaerobic taxa in R2 may be compatible with altered substrate or redox conditions [44,45]. Nevertheless, relative abundance cannot determine absolute bacterial load, strain-level pathogenicity, or metabolite production, and increases in *Escherichia-Shigella*-related reads should not be equated directly with disease. The higher *Candidatus_Arthromitus* signal in R2 should likewise be interpreted cautiously; although segmented filamentous bacteria have been linked to mucosal immune maturation in mammalian models [46], no mucosal immune indicators were measured in the present study.

A plausible working hypothesis is that contrasting energy–protein supply altered the amounts and types of carbohydrate and nitrogenous substrates reaching the hindgut, thereby changing microbial niches and community evenness. The higher measured digestive-enzyme activities in R2 and the distinct fecal community structure may both reflect responses to the same dietary contrast, but the present design does not establish a mechanistic pathway linking enzyme activity to individual microbial taxa or to carcass and meat-quality traits. Protein fermentation can generate ammonia, branched-chain fatty acids, amines, phenols, indoles, and sulfur-containing compounds [47,48,49], but none of these metabolites were measured here. Accordingly, the proposed nutritional–microbial links should be regarded as hypotheses for subsequent testing.

The biological relevance of the factorial results is therefore trait dependent. The ME main effect was most evident for abdominal fat deposition, the CP main effect was evident for several carcass traits, and significant interactions occurred for live weight, liver index, selected meat-quality traits, and most digestive-enzyme activities. These interaction-responsive traits show that increasing one nutrient cannot be assumed to have the same effect at every level of the other nutrient. However, no single dietary combination improved all production, meat-quality, digestive, and microbial outcomes simultaneously, and the present data do not support a unified regulatory mechanism across these endpoints.

The targeted 16S comparison also has limited generalizability. Only two of the nine dietary treatments were sequenced, so the microbiota data cannot estimate the complete main effects of ME and CP or their factorial interaction. Fecal communities may not fully represent the microbiota of the duodenum, jejunum, ileum, or ceca. Moreover, fecal pH, ammonia nitrogen, short-chain and branched-chain fatty acids, other volatile fatty acids, intestinal barrier markers, inflammatory indicators, and microbial absolute abundances were not measured. These omissions limit functional interpretation of the observed taxonomic shifts.

Future studies should combine full-factorial intestinal-segment sampling with metagenomics, metabolomics, targeted fermentation-metabolite measurements, intestinal morphology and barrier assessments, and individual-level linkage of microbial and host phenotypes. Such work is required to determine whether the associations identified here reflect causal nutritional–microbial mechanisms and whether they translate into reproducible production benefits.

## 5. Conclusions

In conclusion, dietary ME and CP exerted trait-specific main and interactive effects on slaughter performance, organ indices, meat quality, intestinal digestive enzyme activity, and fecal microbial community structure in early-weaned gray-feathered King pigeon squabs. Higher ME increased abdominal fat percentage, whereas the effects of ME on live weight and liver index depended on CP level. Dietary CP affected several slaughter traits, with 19% CP supporting higher half-carcass and full-carcass yields. Significant ME × CP interactions were detected for several physiological traits, including live weight, liver index, breast muscle a*, b*, and pH, leg muscle pH, and most measured digestive enzyme activities. The 20% CP + 11.9 MJ/kg ME treatment generally showed higher enzyme activities, but these measurements alone do not demonstrate enhanced secretion or superior production efficiency. In the targeted comparison of two a priori selected contrasting treatments, R1 was characterized by *Lactobacillus*-related taxa, whereas R2 showed higher diversity and greater contributions of Pseudomonadota- and Enterobacterales-related taxa. Because only two treatments were sequenced and fermentation metabolites and intestinal-segment microbiota were not measured, these microbial findings should be interpreted as dietary associations rather than full-factorial or mechanistic evidence.

## Figures and Tables

**Table 1:** Composition and Nutrient Levels of Experimental Diets (air-dry basis).

Items	Groups									
	ME (MJ/kg)	11.90	11.90	11.90	12.20	12.20	12.20	12.50	12.50	12.50
	CP (%)	18	19	20	18	19	20	18	19	20
**Ingredients**										
Corn		52.00	46.10	47.20	47.50	48.60	48.60	50.00	50.20	46.60
Soybean meal		24.50	27.00	31.80	24.00	29.10	33.80	26.20	31.10	34.10
Soybean oil		1.00	1.00	1.50	1.00	1.00	1.00	1.00	1.00	1.50
Pea		10.50	11.90	7.00	12.50	7.50	2.80	8.00	2.90	3.00
Wheat		4.00	4.00	4.00	4.00	4.00	4.00	4.00	4.00	4.00
Wheat bran		1.00	2.00	1.50	2.00	0.80	0.80	0.80	0.80	0.80
Fat powder		0.00	1.00	0.00	2.00	2.00	2.00	3.00	3.00	3.00
Yeast powder		2.00	2.00	2.00	2.00	2.00	2.00	2.00	2.00	2.00
Stone powder		0.80	0.80	0.80	0.80	0.80	0.80	0.80	0.80	0.80
Glucose		0.50	0.50	0.50	0.50	0.50	0.50	0.50	0.50	0.50
Baking soda		0.40	0.40	0.40	0.40	0.40	0.40	0.40	0.40	0.40
NaCl		0.40	0.40	0.40	0.40	0.40	0.40	0.40	0.40	0.40
CaHPO_4_		0.60	0.60	0.60	0.60	0.60	0.60	0.60	0.60	0.60
L-Lys·HCl		0.24	0.24	0.24	0.24	0.24	0.24	0.24	0.24	0.24
DL-Met		0.16	0.16	0.16	0.16	0.16	0.16	0.16	0.16	0.16
Mycotoxin adsorbent		0.10	0.10	0.10	0.10	0.10	0.10	0.10	0.10	0.10
Premix (1)		1.80	1.80	1.80	1.80	1.80	1.80	1.80	1.80	1.80
Total		100.00	100.00	100.00	100.00	100.00	100.00	100.00	100.00	100.00
**Nutrient levels (2)**										
ME (MJ/kg)		11.91	11.91	11.90	12.20	12.20	12.20	12.50	12.50	12.51
CP (%)		17.99	19.00	20.01	17.98	19.00	19.99	18.00	19.01	19.99
EE (%)		3.80	4.46	4.26	5.46	5.48	5.51	6.49	6.52	6.93
Ash (%)		6.78	6.94	7.10	6.78	6.93	7.10	6.78	6.95	7.10
Lys (%)		1.13	1.21	1.26	1.14	1.20	1.26	1.14	1.19	1.26
Met (%)		0.44	0.45	0.47	0.44	0.46	0.47	0.44	0.46	0.47
Ca (%)		0.54	0.55	0.56	0.54	0.55	0.56	0.54	0.55	0.56
TP (%)		0.47	0.49	0.50	0.48	0.48	0.49	0.47	0.48	0.49

(1) The premix provided per kilogram of diet contained vitamin A (VA) 27,000 international units (IU), vitamin D3 (VD3) 90,000 IU, vitamin E (VE) 850 mg, vitamin K3 (VK3) 92 mg, vitamin B2 (VB2) 160 mg, vitamin B6 (VB6) 115 mg, nicotinamide 1050 mg, calcium D-pantothenate 520 mg, D-biotin 2 mg, folic acid 20 mg, Cu 340 mg, Fe 1700 mg, Mn 1700 mg, and Zn 1400 mg. (2) All nutrient levels are calculated values. Crystalline L-Lys·HCl and DL-Met were included at fixed rates in all diets; the diets were not formulated to an identical ideal amino acid profile. Consequently, calculated total Lys and Met varied with ingredient composition and CP level, as reported in Table 1. Abbreviations: ME, metabolizable energy; CP, crude protein; EE, ether extract; Lys, lysine; Met, methionine; Ca, calcium; TP, total phosphorus.

**Table 2:** Effects of Dietary Metabolizable Energy and Crude Protein Levels on Slaughter Performance in Early-Weaned Gray-Feathered King Pigeons.

Items		Live Weight	Slaughter Yield	Half-Carcass Yield	Full-Carcass Yield	Breast Muscle Ratio	Leg Muscle Ratio	Abdominal Fat Percentage
		(g)	(%)	(%)	(%)	(%)	(%)	(%)
CP (%)	ME (MJ/kg)							
18	11.9	459.50 ± 10.59 ^ABab^	87.14 ± 0.70	81.38 ± 1.00	74.81 ± 0.96	26.26 ± 0.7	6.77 ± 0.25	0.66 ± 0.07
	12.2	436.13 ± 8.57 ^Bb^	86.38 ± 0.43	82.04 ± 0.72	74.54 ± 1.06	24.39 ± 0.96	6.58 ± 0.17	1.04 ± 0.15
	12.5	476.25 ± 10.33 ^Aa^	86.85 ± 0.70	81.13 ± 0.96	73.77 ± 1.17	24.68 ± 1.00	6.62 ± 0.18	0.99 ± 0.05
19	11.9	438.00 ± 7.20 ^ABb^	90.29 ± 0.92	84.00 ± 0.99	75.86 ± 0.96	25.07 ± 0.84	6.63 ± 0.16	0.92 ± 0.13
	12.2	457.63 ± 5.10 ^ABab^	90.99 ± 0.35	86.08 ± 0.75	78.35 ± 0.70	26.90 ± 0.49	6.76 ± 0.16	1.28 ± 0.21
	12.5	432.75 ± 5.86 ^Bb^	91.03 ± 0.78	86.23 ± 1.01	78.17 ± 1.45	26.05 ± 0.69	6.68 ± 0.12	1.50 ± 0.23
20	11.9	437.63 ± 12.20 ^ABb^	89.13 ± 0.53	84.77 ± 0.65	77.01 ± 0.78	25.46 ± 0.66	6.37 ± 0.11	1.15 ± 0.12
	12.2	446.88 ± 6.79 ^ABb^	90.14 ± 0.59	82.05 ± 0.75	75.10 ± 0.88	25.71 ± 0.57	6.13 ± 0.14	1.15 ± 0.10
	12.5	456.75 ± 12.66 ^ABab^	89.98 ± 0.58	82.06 ± 1.59	74.55 ± 1.90	25.17 ± 0.73	6.42 ± 0.26	1.24 ± 0.13
**Main effects**								
SEM		5.30	0.37	0.56	0.66	0.44	0.10	0.08
ME (MJ/kg)	11.9	445.04	88.86	83.38	75.89	25.59	6.59	0.91 ^Bb^
	12.2	446.88	89.17	83.39	76.00	25.67	6.49	1.15 ^ABa^
	12.5	455.25	89.29	83.14	75.50	25.30	6.57	1.24 ^Aa^
CP (%)	18	457.29	86.79 ^Bb^	81.52 ^Bb^	74.37 ^Bb^	25.11	6.66 ^a^	0.90 ^Bb^
	19	442.79	90.77 ^Aa^	85.44 ^Aa^	77.46 ^Aa^	26.01	6.69 ^a^	1.23 ^Aa^
	20	447.08	89.75 ^Aa^	82.96 ^Bb^	75.56 ^ABb^	25.45	6.31 ^b^	1.18 ^ABa^
***p*** **-value**								
ME		0.355	0.700	0.939	0.854	0.819	0.773	0.020
CP		0.148	<0.001	<0.001	0.006	0.347	0.020	0.014
ME × CP		0.013	0.689	0.079	0.247	0.190	0.764	0.420

Note: Data are expressed as mean ± SEM. Within a column, values with no superscript or the same superscript do not differ (*p* > 0.05); different lowercase and uppercase superscripts indicate *p* < 0.05 and *p* < 0.01, respectively. ME, metabolizable energy; CP, crude protein; SEM, standard error of the mean.

**Table 3:** Effects of Dietary Metabolizable Energy and Crude Protein Levels on Organ Index in Early-Weaned Gray-Feathered King Pigeons (Unit: %).

Items		Liver Index	Spleen Index	Proventriculus Index	Gizzard Index	Bursa of Fabricius Index
CP (%)	ME (MJ/kg)					
18	11.9	1.86 ± 0.12 ^Bb^	0.08 ± 0.02	0.25 ± 0.01	1.90 ± 0.10	0.20 ± 0.03
	12.2	2.64 ± 0.30 ^Aa^	0.10 ± 0.01	0.27 ± 0.01	1.72 ± 0.06	0.14 ± 0.03
	12.5	2.26 ± 0.16 ^ABab^	0.10 ± 0.02	0.31 ± 0.03	1.76 ± 0.13	0.16 ± 0.01
19	11.9	2.62 ± 0.15 ^Aa^	0.13 ± 0.02	0.30 ± 0.02	1.85 ± 0.09	0.12 ± 0.02
	12.2	2.23 ± 0.16 ^ABab^	0.13 ± 0.04	0.23 ± 0.03	1.86 ± 0.05	0.18 ± 0.02
	12.5	2.46 ± 0.18 ^ABa^	0.15 ± 0.03	0.26 ± 0.01	1.84 ± 0.09	0.18 ± 0.02
20	11.9	2.42 ± 0.12 ^ABa^	0.16 ± 0.02	0.28 ± 0.04	1.66 ± 0.08	0.16 ± 0.01
	12.2	2.18 ± 0.07 ^ABab^	0.10 ± 0.02	0.28 ± 0.04	1.93 ± 0.12	0.16 ± 0.04
	12.5	2.41 ± 0.07 ^ABa^	0.10 ± 0.01	0.28 ± 0.02	1.77 ± 0.07	0.14 ± 0.02
**Main effects**						
SEM		0.09	0.01	0.02	0.05	0.01
ME (MJ/kg)	11.9	2.30	0.12	0.28	1.80	0.16
	12.2	2.35	0.11	0.26	1.84	0.16
	12.5	2.38	0.12	0.28	1.79	0.16
CP (%)	18	2.25	0.10	0.28	1.79	0.17
	19	2.44	0.14	0.26	1.85	0.16
	20	2.34	0.12	0.28	1.79	0.15
***p*** **-value**						
ME		0.825	0.738	0.430	0.804	0.991
CP		0.375	0.077	0.631	0.657	0.726
ME × CP		0.006	0.202	0.287	0.229	0.145

Note: Data are expressed as mean ± SEM. Within a column, values with no superscript or the same superscript do not differ (*p* > 0.05); different lowercase and uppercase superscripts indicate *p* < 0.05 and *p* < 0.01, respectively. ME, metabolizable energy; CP, crude protein; SEM, standard error of the mean.

**Table 4:** Effects of Dietary Metabolizable Energy and Crude Protein Levels on Breast Muscle Quality in Early-Weaned Gray-Feathered King Pigeons.

Items		Water Loss Rate (%)	Shear Force (N)	L*	a*	b*	pH
CP (%)	ME (MJ/kg)						
18	11.9	11.54 ± 2.32	31.40 ± 5.10	28.49 ± 0.76	22.12 ± 0.70 ^Aa^	15.45 ± 0.44 ^Aa^	5.83 ± 0.06 ^Cc^
	12.2	11.08 ± 1.90	25.64 ± 5.54	27.65 ± 0.71	21.42 ± 1.16 ^Aa^	14.15 ± 0.56 ^ABab^	6.09 ± 0.12 ^BCbc^
	12.5	9.55 ± 2.57	24.76 ± 1.79	28.46 ± 1.06	21.91 ± 0.73 ^Aa^	14.29 ± 0.55 ^ABab^	6.09 ± 0.09 ^BCbc^
19	11.9	8.89 ± 1.48	21.98 ± 2.63	26.85 ± 0.65	19.70 ± 1.08 ^ABab^	12.53 ± 0.70 ^Bb^	6.49 ± 0.04 ^Aa^
	12.2	12.58 ± 2.27	23.67 ± 3.23	27.25 ± 1.20	20.91 ± 0.66 ^ABa^	14.54 ± 0.97 ^ABa^	6.31 ± 0.13 ^ABab^
	12.5	11.56 ± 2.62	28.96 ± 4.71	27.25 ± 1.09	20.91 ± 0.49 ^ABa^	13.79 ± 0.61 ^ABab^	6.21 ± 0.11 ^ABCb^
20	11.9	13.76 ± 2.95	27.97 ± 2.76	29.56 ± 0.63	21.44 ± 0.57 ^Aa^	14.86 ± 0.67 ^ABa^	6.21 ± 0.09 ^ABCb^
	12.2	14.52 ± 5.60	28.17 ± 2.67	27.68 ± 0.87	20.80 ± 0.98 ^ABa^	14.32 ± 0.45 ^ABab^	5.86 ± 0.06 ^Cc^
	12.5	8.01 ± 1.79	35.52 ± 4.06	27.63 ± 1.18	17.83 ± 0.91 ^Bb^	12.48 ± 0.46 ^Bb^	6.06 ± 0.06 ^BCbc^
**Main effects**							
SEM		1.65	2.20	0.54	0.48	0.36	0.05
	11.9	11.40	27.12	28.30	21.09	14.28	6.18
ME (MJ/kg)	12.2	12.73	25.83	27.52	21.04	14.33	6.09
	12.5	9.70	29.75	27.78	20.22	13.52	6.12
	18	10.72	27.27	28.20	21.82 ^a^	14.63	6.01 ^Bb^
CP (%)	19	11.01	24.87	27.12	20.51 ^ab^	13.62	6.34 ^Aa^
	20	12.10	30.55	28.29	20.02 ^b^	13.89	6.04 ^Bb^
***p*** **-value**							
ME		0.433	0.443	0.583	0.363	0.209	0.444
CP		0.824	0.195	0.235	0.030	0.128	<0.001
ME × CP		0.631	0.351	0.673	0.047	0.018	0.004

Note: Data are expressed as mean ± SEM. Within a column, values with no superscript or the same superscript do not differ (*p* > 0.05); different lowercase and uppercase superscripts indicate *p* < 0.05 and *p* < 0.01, respectively. ME, metabolizable energy; CP, crude protein; SEM, standard error of the mean. L*, lightness; a*, redness; b*, yellowness.

**Table 5:** Effects of Dietary Metabolizable Energy and Crude Protein Levels on Leg Muscle Quality in Early-Weaned Gray-Feathered King Pigeons.

Items		Water Loss Rate (%)	Shear Force (N)	L*	a*	b*	pH
CP (%)	ME (MJ/kg)						
18	11.9	9.01 ± 1.41	35.11 ± 4.40	34.60 ± 0.97	17.46 ± 1.09	15.71 ± 0.63	6.28 ± 0.05 ^Bc^
	12.2	8.66 ± 1.16	30.63 ± 4.42	32.44 ± 1.74	17.01 ± 1.21	13.68 ± 0.98	6.38 ± 0.04 ^ABbc^
	12.5	6.28 ± 1.19	30.76 ± 2.66	35.06 ± 1.49	14.63 ± 1.34	12.49 ± 0.69	6.36 ± 0.06 ^ABbc^
19	11.9	11.37 ± 2.28	23.01 ± 2.15	34.77 ± 0.94	15.41 ± 0.74	14.20 ± 0.46	6.36 ± 0.04 ^ABbc^
	12.2	10.00 ± 1.94	27.72 ± 3.26	32.63 ± 0.81	18.66 ± 1.22	15.07 ± 1.29	6.47 ± 0.10 ^ABab^
	12.5	11.12 ± 1.69	28.41 ± 2.31	35.77 ± 1.34	14.80 ± 1.23	13.97 ± 0.90	6.39 ± 0.07 ^ABbc^
20	11.9	8.87 ± 0.83	27.31 ± 3.16	35.54 ± 0.93	14.99 ± 0.70	13.98 ± 0.37	6.57 ± 0.04 ^Aa^
	12.2	9.86 ± 1.84	29.06 ± 2.37	35.23 ± 2.11	13.81 ± 1.63	11.89 ± 1.26	6.33 ± 0.04 ^ABbc^
	12.5	8.34 ± 0.73	32.24 ± 2.91	37.13 ± 1.93	14.11 ± 0.94	14.32 ± 1.01	6.37 ± 0.05 ^ABbc^
**Main effects**							
SEM		0.89	1.83	0.83	0.67	0.52	0.03
ME (MJ/kg)	11.9	9.75	28.48	34.97	15.95	14.63	6.40
	12.2	9.51	29.14	33.43	16.49	13.55	6.40
	12.5	8.58	30.47	35.99	14.51	13.59	6.37
CP (%)	18	7.98	32.17	34.03	16.37	13.96	6.34
	19	10.83	26.38	34.39	16.29	14.41	6.41
	20	9.02	29.54	35.97	14.30	13.40	6.42
***p*** **-value**							
ME		0.617	0.737	0.099	0.105	0.256	0.822
CP		0.079	0.079	0.223	0.054	0.387	0.164
ME × CP		0.762	0.464	0.963	0.232	0.076	0.015

Note: Data are expressed as mean ± SEM. Within a column, values with no superscript or the same superscript do not differ (*p* > 0.05); different lowercase and uppercase superscripts indicate *p* < 0.05 and *p* < 0.01, respectively. ME, metabolizable energy; CP, crude protein; SEM, standard error of the mean. L*, lightness; a*, redness; b*, yellowness.

**Table 6:** Effects of Dietary Metabolizable Energy and Crude Protein Levels on Intestinal Digestive Enzyme Activity in Early-Weaned Gray-Feathered King Pigeons.

Items		Duodenum				Jejunum			
		Trypsin (U/mgprot)	Chymotrypsin (U/mgprot)	Lipase (U/gprot)	α-Amylase (U/mgprot)	Trypsin (U/mgprot)	Chymotrypsin (U/mgprot)	Lipase (U/gprot)	α-Amylase (U/mgprot)
CP (%)	ME (MJ/kg)								
18	11.9	263.39 ± 10.81 ^CD^	269.33 ± 12.08	668.10 ± 8.84 ^E^	1.78 ± 0.06 ^B^	161.77 ± 6.28 ^BC^	232.05 ± 9.11 ^D^	575.73 ± 8.40 ^D^	1.14 ± 0.05 ^DE^
	12.2	228.24 ± 7.34 ^DE^	243.33 ± 11.15	697.62 ± 9.29 ^DE^	1.37 ± 0.04 ^CD^	171.37 ± 5.11 ^B^	250.71 ± 10.85 ^CD^	581.03 ± 8.21 ^D^	1.09 ± 0.04 ^DE^
	12.5	165.17 ± 5.62 ^F^	219.79 ± 9.33	606.84 ± 11.71 ^F^	1.22 ± 0.05 ^D^	104.55 ± 2.46 ^E^	163.02 ± 5.42 ^E^	528.84 ± 8.00 ^E^	0.98 ± 0.05 ^E^
19	11.9	354.88 ± 13.38 ^AB^	364.37 ± 12.43	766.44 ± 9.76 ^B^	2.34 ± 0.11 ^A^	193.33 ± 2.29 ^A^	281.91 ± 7.52 ^BC^	710.06 ± 10.95 ^B^	1.46 ± 0.05 ^B^
	12.2	219.80 ± 8.81 ^E^	326.43 ± 14.65	712.20 ± 10.01 ^D^	1.64 ± 0.08 ^BC^	146.10 ± 5.57 ^CD^	247.78 ± 12.89 ^CD^	664.38 ± 10.06 ^C^	1.29 ± 0.06 ^BCD^
	12.5	334.80 ± 11.34 ^B^	328.59 ± 14.83	719.82 ± 10.27 ^CD^	1.56 ± 0.06 ^BC^	128.00 ± 4.37 ^D^	235.38 ± 8.84 ^D^	566.16 ± 10.45 ^DE^	1.19 ± 0.06 ^CD^
20	11.9	388.94 ± 4.27 ^A^	393.11 ± 6.99	830.26 ± 4.87 ^A^	2.52 ± 0.11 ^A^	212.50 ± 6.10 ^A^	325.21 ± 7.09 ^A^	813.00 ± 11.80 ^A^	1.68 ± 0.05 ^A^
	12.2	349.56 ± 9.92 ^B^	358.92 ± 10.06	755.02 ± 11.35 ^BC^	2.23 ± 0.10 ^A^	196.89 ± 5.52 ^A^	288.99 ± 6.79 ^B^	715.50 ± 11.88 ^B^	1.40 ± 0.06 ^BC^
	12.5	273.12 ± 13.99 ^C^	333.63 ± 10.57	675.43 ± 6.15 ^E^	1.50 ± 0.05 ^BCD^	147.81 ± 5.53 ^C^	263.26 ± 12.57 ^BCD^	559.36 ± 10.44 ^DE^	1.20 ± 0.05 ^CD^
**Main effects**									
SEM		5.77	6.69	5.42	0.04	2.89	5.39	5.84	0.03
ME (MJ/kg)	11.9	335.73 ^A^	342.27 ^A^	754.93 ^A^	2.21 ^A^	189.20 ^A^	279.72 ^A^	699.59 ^A^	1.43 ^A^
	12.2	265.87 ^B^	309.56 ^B^	721.61 ^B^	1.75 ^B^	171.45 ^B^	262.50 ^A^	653.63 ^B^	1.26 ^B^
	12.5	257.69 ^B^	294.00 ^B^	667.36 ^C^	1.43 ^C^	126.78 ^C^	220.56 ^B^	551.45 ^C^	1.12 ^C^
CP (%)	18	218.94 ^C^	244.15 ^B^	657.52 ^C^	1.46 ^C^	145.89 ^B^	215.26 ^C^	561.86 ^C^	1.07 ^C^
	19	303.16 ^B^	339.80 ^A^	732.82 ^B^	1.85 ^B^	155.81 ^B^	255.03 ^B^	646.87 ^B^	1.31 ^B^
	20	337.20 ^A^	361.89 ^A^	753.57 ^A^	2.08 ^A^	185.73 ^A^	292.49 ^A^	695.95 ^A^	1.43 ^A^
***p*** **-value**									
ME		<0.001	<0.001	<0.001	<0.001	<0.001	<0.001	<0.001	<0.001
CP		<0.001	<0.001	<0.001	<0.001	<0.001	<0.001	<0.001	<0.001
ME × CP		<0.001	0.725	<0.001	<0.001	<0.001	0.001	<0.001	0.047

Note: Data are expressed as mean ± SEM. Within a column, values with no superscript or the same superscript do not differ (*p* > 0.05); different uppercase superscripts indicate *p* < 0.01. ME, metabolizable energy; CP, crude protein; SEM, standard error of the mean.

**Table 7:** Comparison of fecal microbial alpha diversity indices between the R1 and R2 groups.

Items	R1 Group	R2 Group
Shannon index	1.61 ± 0.30 ^B^	2.55 ± 0.72 ^A^
Simpson index	0.51 ± 0.09 ^B^	0.70 ± 0.09 ^A^
Pielou_e index	0.27 ± 0.04 ^B^	0.38 ± 0.07 ^A^
Chao1 index	61.19 ± 14.30	103.72 ± 52.21
Observed features	60.63 ± 13.55	103.63 ± 52.18

Note: Data are expressed as mean ± SD. R1, 18% CP + 12.5 MJ/kg ME; R2, 20% CP + 11.9 MJ/kg ME. Within a row, values with different uppercase superscripts indicate *p* < 0.01. ME, metabolizable energy; CP, crude protein; SD, standard deviation.

## Data Availability

The original contributions presented in this study are included in the article. Further inquiries can be directed to the corresponding author.
